# Digitally Fabricated Orbital Prosthesis for the Rehabilitation of Orbital Defects Following Squamous Cell Carcinoma (SCC)

**DOI:** 10.7759/cureus.83489

**Published:** 2025-05-05

**Authors:** Ullash Kumar, Lokanath Garhnayak, Tapan K Patro, Angurbala Dhal, Anaida Acharya, Sandesh Belkhede, Subhendu K Panda

**Affiliations:** 1 Prosthodontics, SCB Dental College and Hospital, Cuttack, IND; 2 Prosthodontics and Crown &amp; Bridge, SCB Dental College and Hospital, Cuttack, IND

**Keywords:** dental, maxillofacial, orbital, prosthesis, rehabilitation

## Abstract

The rehabilitation of facial defects is a challenging effort that calls for a customized approach tailored to each patient. The disfiguration resulting from the loss of an eye can lead to substantial physical and emotional issues. There are several treatment options available. Fabrication of a spectacle-retained digitally designed silicone orbital prosthesis has been detailed in this case report. This report highlights the utilization of digital dentistry to simplify and enhance the treatment protocol.

## Introduction

The loss of part of the face can have physical, social, and psychological impacts on those affected [1]. Maxillofacial prostheses restore and replace stomatognathic and associated facial structures with artiﬁcial substitutes. The goal is to enhance the patient's appearance, maintain the health of the remaining structures, and ultimately promote physical and mental well-being. There is a high demand for these prostheses. According to a five-year survey conducted in the Maxillofacial Unit of the School of Oral Health Sciences at the University of the Witwatersrand, Johannesburg, South Africa, approximately 25% of maxillofacial defects are related to ocular defects [2].

The loss of an eye can result from various causes, including congenital defects, severe injury, tumors, or the need for further medical intervention. Surgical options for managing this condition may include evisceration, enucleation, or exenteration, depending on the severity. Evisceration involves removing the inner contents of the eye while keeping the outer parts intact. Enucleation is the removal of the entire eye and a part of the optic nerve from the socket. Exenteration is the complete removal of the eye socket, often performed to treat malignant orbital tumors. After exenteration, patients may require an orbital prosthesis for rehabilitation due to the extensive nature of the surgical procedure.

Early orbital prosthesis restoration dates back to the fourth dynasty in Egypt. Excavations of graves have uncovered evidence of using precious stones, earthenware, copper, gold, and enameled bronze to replace eyes in shrunken eye sockets. Paré also utilized glass and porcelain for eyes, representing a significant advancement [3]. Methyl methacrylate prosthesis became popular due to its superior strength and the ability to be modified in shape and size. Flexible materials like silicone became advantageous when the defect extended beyond the orbital area and involved movable tissue beds.

Veerareddy et al. [4] proposed an economical and simple way of making an orbital prosthesis that used economical material (irreversible hydrocolloid) with a modified impression tray to block the overflow of material on the patient’s face. Artopoulou et al. [5] introduced a method for creating artificial eye prosthesis using digital photography. Nevertheless, creating an orbital prosthesis is still challenging in numerous situations.

Today, many people use digital techniques, which offer numerous benefits. By replacing conventional, manually intensive procedures, digital methods can enhance patient precision, improve quality of life, and yield better treatment outcomes, all while optimizing health services. In this case report, we discuss the digital fabrication of a spectacle-retained silicone orbital prosthesis using CT imaging and digital planning. We chose a CT scan over a facial scan because the information obtained from the upper half of the face was sufficient for fabricating the orbital prosthesis. Additionally, in a country like India, CT scanners are more readily available than facial scanners.

## Case presentation

A 40-year-old male patient presented to the Department of Prosthodontics and Crown & Bridge, SCB Dental College and Hospital with a missing left eye. The patient provided a history of eye exenteration due to squamous cell carcinoma (SCC) eight months prior. He did not have any systemic diseases.

As the defect had minimal anatomical undercut and the patient was not interested in any additional surgical procedures to aid in retention, a spectacle-retained prosthesis was planned.

**Figure 1:**
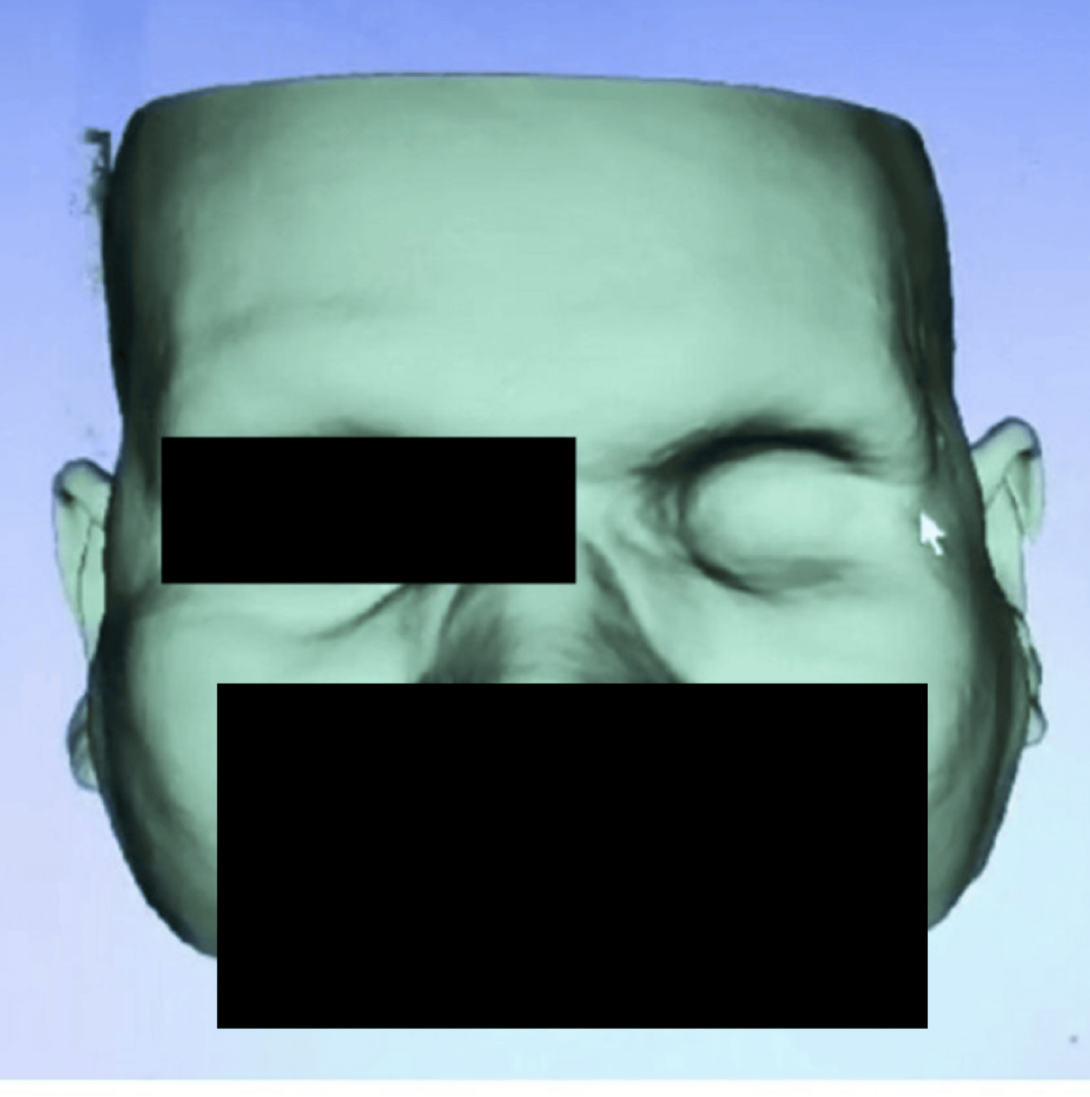
Digitally obtained impression of the left orbit using CT scan.

After the external examination, the impression of the orbit was taken digitally with the help of CT scanning of the orbit region (Figure 1).

**Figure 2:**
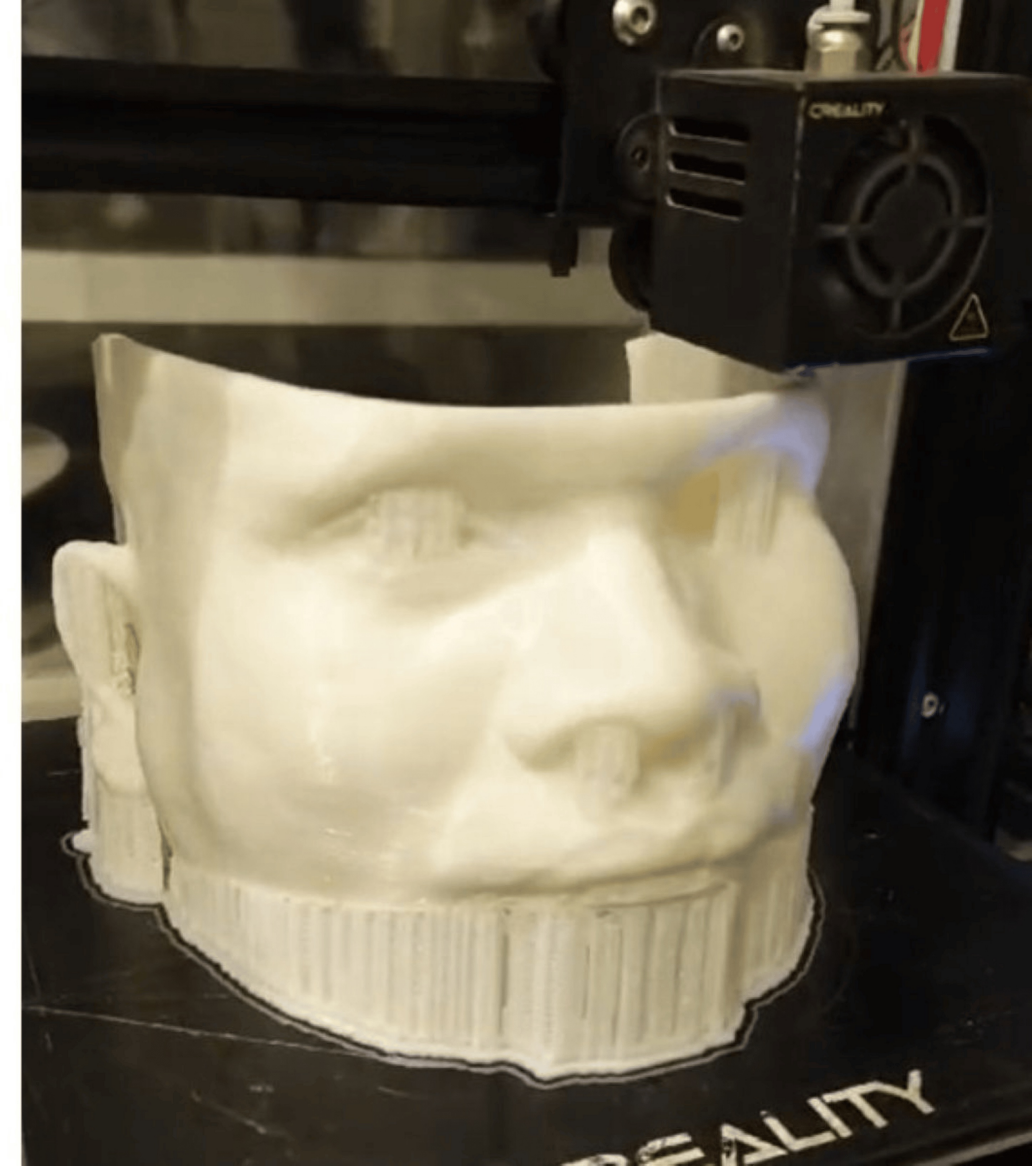
Patient model 3D printed using polylactic acid (PLA) material.

This scanned image was then sent to the laboratory, where 3D printing of the patient model was carried out (Figure 2).

**Figure 3:**
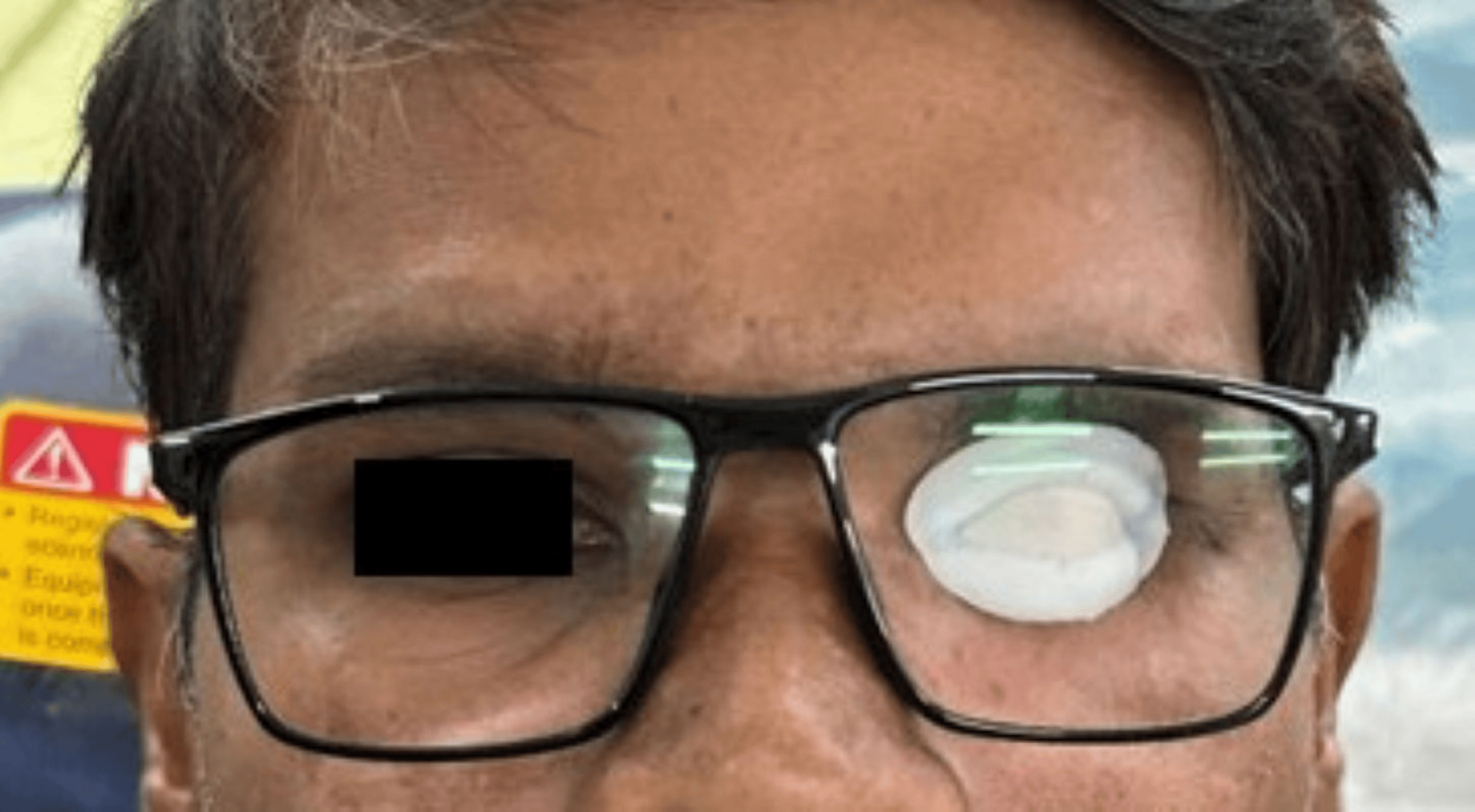
Template placed on the patient, and the iris positioning completed.

Then, using digital software, a template was fabricated to mimic the contours of the opposite eye. At the next appointment, the template was placed in the defect region to check the fit, and iris positioning was performed, as shown in Figure 3.

The selected pair of spectacles was tried on the patient, and the patient's approval was obtained. High-definition digital photographs of the adjacent eye were also taken and sent to the lab for reference, along with the template with the iris positioned.

**Figure 4:**
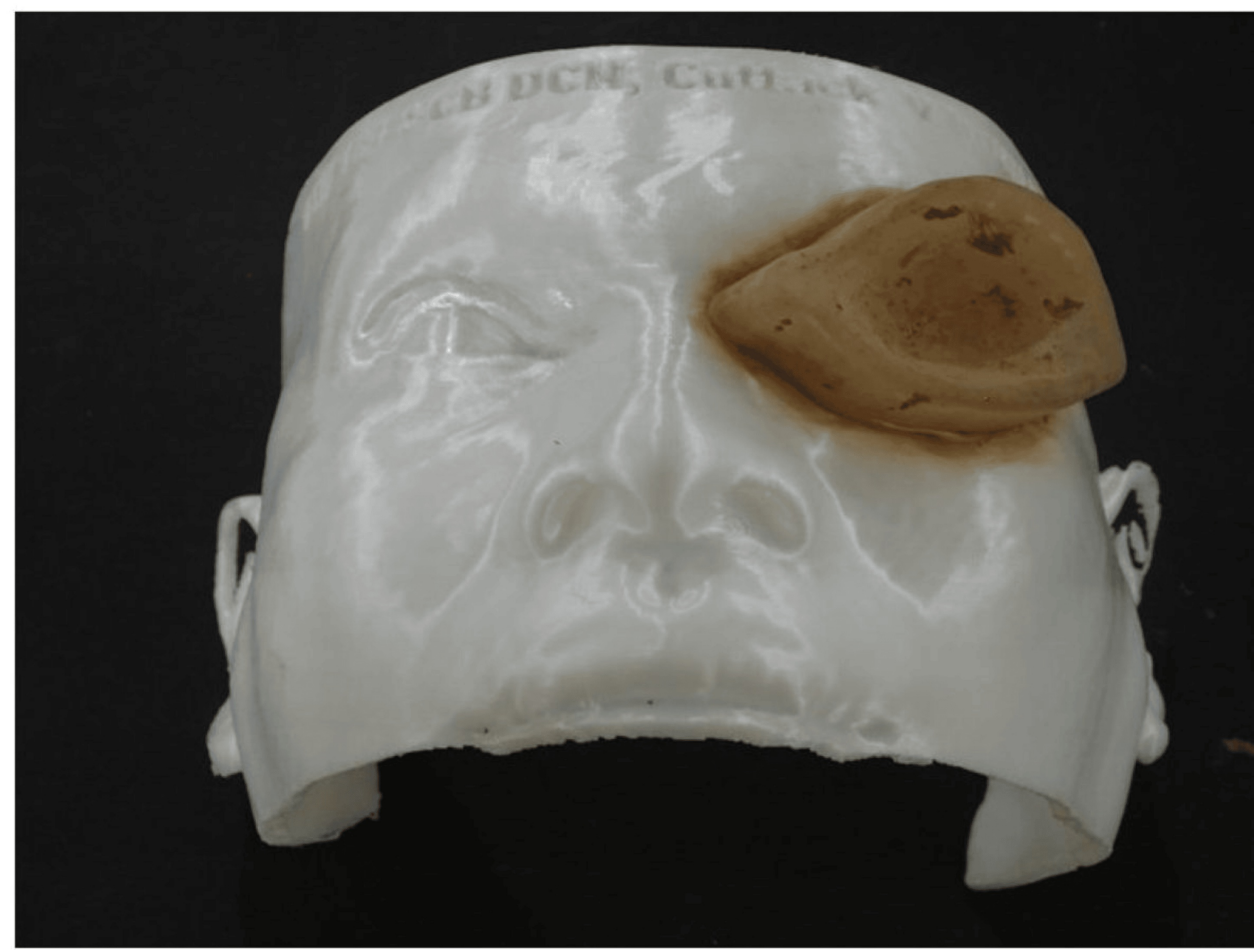
3D-printed resin mold used for fabricating the silicone prosthesis.

The template was scanned and superimposed over the previous digital model. Further enhancement of the contour was done, and the ocular part was designed by the photographs of the adjacent eyeball. The ocular part of the prosthesis was 3D printed first and placed onto the 3D printed patient model. To fabricate the silicone part of the orbital prosthesis, a mold was created based on the planned contour from the digital image. This mold was placed over the model to form the silicone orbital prosthesis (Figure 4).

**Figure 5:**
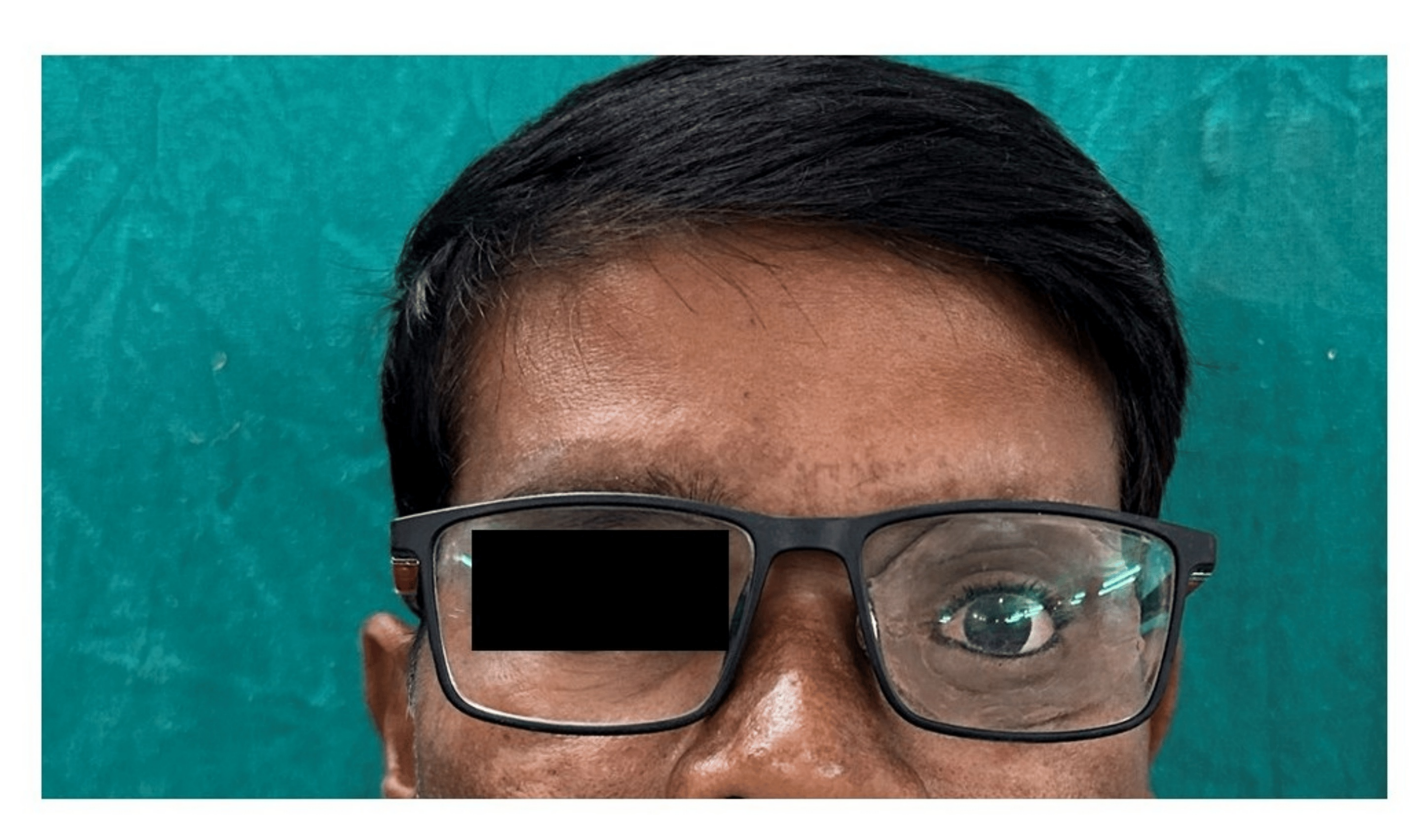
Digitally fabricated silicone spectacle-retained orbital prosthesis in situ.

The silicone was mixed with intrinsic stains and cured according to the manufacturer’s instructions. Eyelashes were glued to the prosthesis. Extrinsic stains were applied to mimic the patient's skin tone and cured according to the manufacturer’s instructions. The orbital prosthesis was attached to the previously selected spectacles using orthodontic wire to allow for ease of movement and proper seating of the prosthesis into the defect. The final prosthesis was then tried on the patient’s face (Figure 5).

The patient was highly satisfied with the final prosthesis. Also, with the help of this digital workflow, the number of appointments was reduced significantly. This also expedited the treatment process and enhanced patient comfort.

## Discussion

Constructing an orbital prosthesis involves several challenges, including creating a functional model without compressing the tissue, positioning the ocular part to match the remaining eye, replicating the shape and structure of the periorbital tissues, determining the correct gaze and interlid opening, and achieving a suitable color match. Additionally, important considerations include selecting the materials and methods for fabrication and determining how the prosthesis will be secured in place.

Most of these challenges can be solved with the help of digitization. Instead of recording the patient’s anatomy with a conventional impression where some amount of compressibility can be expected, this case report showed the use of a CT scan of the patient to create a model with complete accuracy [6]. According to Nightingale et al., a treatment's efficacy is related to its ability to replicate the patient's original anatomy and physiological positive effects [7].

The process of contouring the lost soft tissue and matching the ocular segment was also improved through the use of digital design software.

Orbital prostheses can be made from acrylic or silicone. While acrylic prostheses are simpler to fabricate and repair, silicone prostheses offer the added benefits of being lightweight and having superior adaptability. Hence, in this case, we decided to fabricate a silicone orbital prosthesis with the help of digital software.

The orbital prosthesis can be retained in place by using adhesives, attaching it to eyeglasses, or fitting it into hard or soft tissue undercuts. Currently, there are four types of tissue adhesives: fibrin sealants, collagen-based sealants, synthetic polymer-based materials, and protein-based sealants. Many people prefer using osseointegrated implants because they provide better retention than other methods [6]. However, not everyone can use these implants due to factors like overall health and financial limitations. In the present case, the patient was not psychologically prepared for any surgical procedure; therefore, anatomical undercuts and spectacles were used for retention.

Creating digital designs for orbital prostheses remains a challenge. Additionally, traditional human involvement is still necessary in certain stages of the manufacturing process, which has hindered a complete shift to digital fabrication for maxillofacial prostheses [8]. As a result, there have been suggestions to adopt a hybrid approach, such as using photogrammetry to scan the defect and then employing 3D printing to fabricate the prosthesis [9]. This approach combines the advantages of both analog and digital methods. Photogrammetry is a method for creating 3D digital models from images taken from different angles. The study conducted by Bansod et al. also evaluated the accuracy differences between CT scans and photogrammetry [9]. Although the CT scan image had a much higher resolution, the accuracy level was comparable. Therefore, in a rural setting, photogrammetry can also be used to obtain patient data. This hybrid approach, combining analog and digital methods, may help mitigate the drawbacks of a fully digital workflow, such as the higher cost of prosthesis fabrication and the associated learning curve.

There is no ideal recipe or methodology to fabricate a maxillofacial prosthesis. The treatment flowchart depends on the facilities available and the expertise of the operator.

## Conclusions

The quality and result of digitally designed prosthesis depend significantly on the data acquisition method and the type of product being designed - whether it's a template, mold, or directly printed prosthesis. Using a template allowed for a trial phase before creating the final prosthesis. The authors favored digitally designing the mold for the prosthesis, as it enabled manual color matching and aesthetic contouring in this case.

The workflow outlined in this case study demonstrates the ability to create an orbital prosthesis that achieves excellent facial symmetry, matches skin tone, and provides a comfortable fit. The stored data enable efficient digital adjustments to perfect the prosthesis without the need for the patient to be physically present. By utilizing CT mapping, 3D printing for templates and molds, and silicone casting, this method could enhance the availability of orbital prostheses worldwide.
